# Protective effect of synbiotic combination of *Lactobacillus plantarum SC-5* and olive oil extract tyrosol in a murine model of ulcerative colitis

**DOI:** 10.1186/s12967-024-05026-9

**Published:** 2024-03-25

**Authors:** Fazheng Yu, Xueyu Hu, HongLin Ren, Xiaoxu Wang, Ruoran Shi, Jian Guo, Jiang Chang, Xiaoshi Zhou, Yuanyuan Jin, Yansong Li, Zengshan Liu, Pan Hu

**Affiliations:** 1 State Key Laboratory for Diagnosis and Treatment of Severe Zoonotic Infectious Diseases, Key Laboratory for Zoonosis Research of the Ministry of Education, Institute of Zoonosis, and College of Veterinary Medicine, Jilin University, Changchun, 130062 China; 2 Institute of Special Animal and Plant Sciences of Chinese Academy of Agricultural Sciences, Changchun, 130112 Jilin China; 3 Jilin Academy of Animal Husbandry and Veterinary Sciences, Changchun, 130062 China

**Keywords:** Ulcerative colitis, Dextran sulfate sodium, Tyrosol, *Lactobacillus plantarum*, NF-κB, MAPK, Intestinal microbiota

## Abstract

**Background:**

Ulcerative colitisis (UC) classified as a form of inflammatory bowel diseases (IBD) characterized by chronic, nonspecific, and recurrent symptoms with a poor prognosis. Common clinical manifestations of UC include diarrhea, fecal bleeding, and abdominal pain. Even though anti-inflammatory drugs can help alleviate symptoms of IBD, their long-term use is limited due to potential side effects. Therefore, alternative approaches for the treatment and prevention of inflammation in UC are crucial.

**Methods:**

This study investigated the synergistic mechanism of *Lactobacillus plantarum* SC-5 (SC-5) and tyrosol (TY) combination (TS) in murine colitis, specifically exploring their regulatory activity on the dextran sulfate sodium (DSS)-induced inflammatory pathways (NF-κB and MAPK) and key molecular targets (tight junction protein). The effectiveness of 1 week of treatment with SC-5, TY, or TS was evaluated in a DSS-induced colitis mice model by assessing colitis morbidity and colonic mucosal injury (n = 9). To validate these findings, fecal microbiota transplantation (FMT) was performed by inoculating DSS-treated mice with the microbiota of TS-administered mice (n = 9).

**Results:**

The results demonstrated that all three treatments effectively reduced colitis morbidity and protected against DSS-induced UC. The combination treatment, TS, exhibited inhibitory effects on the DSS-induced activation of mitogen-activated protein kinase (MAPK) and negatively regulated NF-κB. Furthermore, TS maintained the integrity of the tight junction (TJ) structure by regulating the expression of zona-occludin-1 (ZO-1), Occludin, and Claudin-3 (*p* < 0.05). Analysis of the intestinal microbiota revealed significant differences, including a decrease in *Proteus* and an increase in *Lactobacillus*, *Bifidobacterium*, and *Akkermansia*, which supported the protective effect of TS (*p* < 0.05). An increase in the number of *Aspergillus* bacteria can cause inflammation in the intestines and lead to the formation of ulcers. *Bifidobacterium* and *Lactobacillus* can regulate the micro-ecological balance of the intestinal tract, replenish normal physiological bacteria and inhibit harmful intestinal bacteria, which can alleviate the symptoms of UC. The relative abundance of *Akkermansia* has been shown to be negatively associated with IBD. The FMT group exhibited alleviated colitis, excellent anti-inflammatory effects, improved colonic barrier integrity, and enrichment of bacteria such as *Akkermansia* (*p* < 0.05). These results further supported the gut microbiota-dependent mechanism of TS in ameliorating colonic inflammation.

**Conclusion:**

In conclusion, the TS demonstrated a remission of colitis and amelioration of colonic inflammation in a gut microbiota-dependent manner. The findings suggest that TS could be a potential natural medicine for the protection of UC health. The above results suggest that TS can be used as a potential therapeutic agent for the clinical regulation of UC.

**Supplementary Information:**

The online version contains supplementary material available at 10.1186/s12967-024-05026-9.

## Introduction

Ulcerative colitis (UC) is a chronic inflammatory disease that primarily affects the intestinal tract, specifically the mucosal and submucosal regions [1]. It typically originates from inflammation of the rectal mucosa and can extend proximally to involve the colon and other areas [2, 3]. UC is classified as a form of inflammatory bowel disease (IBD) characterized by chronic, nonspecific, and recurrent symptoms [4]. Common clinical manifestations of UC include diarrhea, fecal bleeding, and abdominal pain [5]. The prevalence of UC has been increasing globally, likely due to changes in diet and lifestyle [6]. Globally, more than 10 million people suffer from IBD [7] The reported global prevalence of UC ranges from 2.42 to 298.5/100,000, with the highest incidence reported in North America and Northern Europe [8]. Although anti-inflammatory drugs such as non-steroidal anti-inflammatory drugs (NSAIDs) and selective COX-2 inhibitors can reduce the production of inflammatory mediators, helping to control the development of ulcerative colitis and alleviate symptoms [9]. However, the long-term use of antibiotics, aminosalicylates, corticosteroids, and immunosuppressants may lead to a series of adverse reactions, such as allergic reactions, metabolic disorders, and liver diseases [10]. These adverse reactions may have an impact on the health of patients. Therefore, alternative approaches for the treatment and prevention of inflammation in UC are crucial.

Although the exact pathogenesis of UC remains unclear, studies have indicated that genetic predisposition, environmental factors, immune stimulation, and intestinal microorganisms play significant roles in its development [11, 12]. Oxidative stress has also been implicated in exacerbating UC by promoting the production of reactive oxygen species (ROS) by neutrophils, leading to increased inflammation and damage to the intestinal mucosa [13]. Hence, the exploration of safe and natural antioxidant agents for the treatment of UC is of particular importance [14]. Natural antioxidant products are substances that, upon ingestion, provide beneficial effects on human health [15]. Recent studies have shown that specific natural antioxidant products can modulate the intestinal microbiota, thereby aiding in the treatment and prevention of colitis [16]. Research has reported that combining natural antioxidant products with probiotics can enhance the effectiveness of alleviating UC symptoms [17, 18]. However, there are few researches on the combination of natural antioxidant products and probiotics for protecting against UC. Based on our earlier research, we found that the natural antioxidant product TY and the probiotic SC-5 possess strong anti-inflammatory and antioxidant capabilities [19, 20]. For this purpose, we combined the natural antioxidant product TY with the probiotic SC-5 to explore the protective mechanisms of TS in ulcerative colitis mice.

To investigate the protective effect of TS on UC, we hypothesized that oral supplementation of TS can modulate the gut microbiota to alleviate colitis, reduce host inflammation (NF-κB and MAPK signaling pathways), and oxidative stress levels, thus protecting against the effects of UC. Additionally, high-throughput microbial 16SrRNA gene sequencing was performed to analyze the impact of TS on the gut microbiota and its relationship with UC. Our findings highlight the potential of TS as a candidate drug for the treatment and prevention of colitis, and provide new insights into the development of probiotics combined with natural antioxidant products. This study contributes to our understanding of the mechanisms involved in UC and offers a promising therapeutic approach targeting the gut microbiota.

## Materials and methods

### Chemicals

Dextran Sulfate Sodium Salt (DSS) (Cat#9011-18-1) was purchased from MP Biomedicals (Irvine, CA). Specific antibodies including ZO-1 (1:1000; #AF5145; RRID:AB_2837631), Occludin (1:1000; #DF7504; RRID:AB_2841004), Claudin-3 (1:1000; #AF0129; #RRID:AB_2833313), p65 (1:1000; #BF8005:AB_2846809), p-p65 (1:1000; AB_2834435:AF2006), IKBα (1:1000; #AF5002:AB_2834792), p-IKBα (1:1000; #AB_2834433:AF2002), ERK (1:1000; #AB_2833336:AF0155), p-ERK (1:1000; #AB_2834432:AF1015), JNK (1:1000; #AF6318:AB_2835177), p-JNK (1:1000; #AF3318:AB_2834737), p38 (1:1000; #BF8015:Q16539), p-p38 (1:1000; #AF4001:AB_2835330) and GAPDH (1:1000; #AF7021; AB_2839421) were purchased from Affinity Biosciences (OH, USA). Tumor necrosis factor (TNF)-α (Cat# 430915), interleukin (IL)-1β (Cat# 432615), and interleukin (IL)-6 (Cat# 431307) enzyme-linked immunosorbent assay (ELISA) kits were obtained from Biolegend (San Diego, California, USA). Myeloperoxidas (MPO) (A044-1-1), Superoxide Dismutase (T-SOD) (A001-3-2), Malondialdehyde (MDA) (A003-1-2), Glutathione Peroxidase (GSH-PX) (A005-1-2), and Catalase (CAT) (A007-1-1) assay kit was bought from Nanjing Jiancheng Bioengineering Institute (Nanjing, China).

### Bacterial liquid preparation

The *Lactobacillus plantarum SC-5* strain was obtained from the Key Laboratory of Human-Veterinary Diseases, Jilin University, Ministry of Education, China (JUIZ). As previously described methods [19], the SC-5 strain was cultured in De Man, Rogosa, and Sharpe (MRS) medium. The culture was incubated at 37 °C and 180 rpm in a shaking incubator for 24 h. MRS medium is a commonly used medium for culturing lactic acid bacteria. After the incubation period, the bacterial culture was collected by centrifugation at 4000 rpm/min for 10 min. Centrifugation helps to separate the bacterial cells from the culture medium. The collected bacteria were washed three times with phosphate-buffered saline (PBS) solution. Washing with PBS helps to remove any residual culture medium or impurities. After the washing steps, the bacteria were resuspended in PBS to achieve a concentration of 1.0 × 10^10^ colony-forming units per milliliter (CFU/mL). This concentration ensures that the bacterial liquid is sufficiently concentrated for further experiments or applications.

### Animal model of DSS-induced UC

The immune system characteristics of C57BL/6 mice are similar to humans, making them a good representative model for studying IBD [21]. DSS, by inducing damage to the colonic mucosa and eliciting inflammatory responses in mice, effectively simulates the pathophysiological process of UC [22]. Therefore, it is widely used in the study of IBD, particularly as an experimental animal model for UC. Male C57BL/6 mice (6–8 weeks old, 22–24 g) were purchased from the Experimental Animal Center of Jilin University (Jilin, China). All experimental procedures were performed according to the guidelines of the Institutional Animal Care and Use Committee of Jilin University (No. SY202010004). Mice were kept in a specific pathogen-free environment at a temperature of (24 ± 1 °C) for 1 week prior to the formal experiment. Mice were randomized into five groups, (n = 9/group), an untreated normal control group (PBS treatment only), a DSS control group (DSS + treatment only), another SC-5 group (DSS-treatment and SC-5 1.0 × 10^10^ CFU/kg/day), and a TY group (DSS treatment and tyrosol (20 mg/kg/day)) [20]. And one TS group (DSS treatment with tyrosol (20 mg/kg/day) in combination with SC-5, after 7 days we induced acute colitis by drinking water with DSS at a dose of 3% (w/V) for 7 days, mice were sacrificed on day 14, and colon length was measured.

For fecal transplantation, each group of fresh feces was pooled and homogenized, diluted in sterile saline at a final concentration of 50 mg feces/mL. The samples were centrifuged at 100×*g* for 2 min, and the resulting supernatant was filtered through a 70 μm filter before being utilized for FMT treatment. After drinking water containing 3% DSS for 7 days, each mouse was given a total of 100 μL of FMT per day by oral gavage [23].

### Disease Activity Index (DAI) score

DAI is a commonly used measure for assessing the severity of IBD. The DAI is a method for assessing the activity of inflammatory diseases, scoring based on three different clinical parameters: percentage of weight loss, stool consistency, and the presence of fecal blood. The DAI score (0–12) was the total score of above three parameters, body weight, fecal morphology and fecal occult blood were recorded during the experiment. The DAI was scored based on the mean scores of weight change, stool consistency, and bleeding volume in a previous scoring system [24]. Measurements were performed in six replicates.

### Histopathological analysis

The proximal colon tissue was fixed in 4% formaldehyde solution. The colon segments were embedded in paraffin using standard procedures, and the sections were stained with H&E and AB-PAS. Histological sections were scored for pathological damage using established scoring criteria (n = 9) [4]. Histological scoring was performed in a blinded fashion by two pathologists. Colitis was scored based on the combined scores of inflammatory cell infiltration (score 0–4), ulceration (score 0–4) and area of crypt distortion (score 0–4).

### Measurement of pro-inflammatory cytokines level

Colonic tissue was scissored and homogenized in phosphate solution (PBS = 7.4), and the lysate was centrifuged at 12,000×*g*, 4 °C for 20 min, and the supernatant was aspirated. TNF-α, IL-1β, and IL-6 in tissues were detected using ELISA kits (ELISA MAX Deluxe Sets; BioLegend), with optical density measured at 450 nm and background measured at 570 nm subtracted [18]. The data may be affected by the incubation time, incubation temperature, plate washing times, spectrophotometer wavelength, and the nature of different antibodies. There may be some deviation in the OD value, but the data have been counted three times and the average value is taken.

### Measurement of oxidative stress level

Commercial kits (Nanjing Jiancheng Bioengineering Institute, Nanjing, China) were used to detect representative oxidoreductases such as MPO, T-SOD, CAT, T-AOC, GSH-PX and MDA [21]. The data may be affected by the incubation time, incubation temperature, plate washing times, spectrophotometer wavelength, and the nature of different antibodies. There may be some deviation in the OD value, but the data have been counted three times and the average value is taken.

### Western blot analysis

Colon tissue was homogenized using standard RIPA buffer (Invitrogen) supplemented with protease inhibitor cocktail (Roche) and the total protein concentration of the supernatant of the colon homogenate was determined using the BCA Protein Assay (Solarbio, China) kit. Protein samples (30 µg) were separated by electrophoresis on 15% SDS polyacrylamide gel, and then the samples were placed on nitrocellulose membrane (PVDF). To measure the expression of ZO-1, Occludin, Claudin-3, p38, p-p38, JNK, p-JNK, ERK, p-ERK, NF-κB p65, p-p65, IκBα, p-IκBα, and GADPH. The bands were visualized with a Bio-Rad ChemiDoc™ MP. Quantitative analysis of Western blot bands was performed using ImageJ software 6.0 (National Institutes of Health, Bethesda, MD, USA) [25].

### Immunohistochemistry

Immunohistochemistry was performed as previously described [25]. To probe the expression level of localization-quantified protein in colon tissue, sections were incubated overnight at 4 °C in primary antibody (anti-ZO-1 1:200). The sections were then washed with PBS and incubated for staining with HRP-labeled anti-Rabbit IgG (1:1000). After washing, the sections were treated with DAB staining solution, and the sections were placed under the microscope to detect the image with 100× magnification, and the colon morphology was detected and compared. The relative intensities of stained proteins were determined by auto-mated image analyses in three to five randomly selected fields for every sample. ImageJ 6.0 software (National Institutes of Health, Bethesda, MD, USA) was used to obtain the average fluorescence intensity (mean gray value) of the fluorescence channel.

### Microbial sequencing of cecal content

Samples of cecal contents were frozen in liquid nitrogen immediately after removal and then transferred to − 80 °C. Subsequently, bacterial DNA was extracted from caecal contents of Control, DSS, SC-5, TY and TS groups, and the V3–V4 region of 16s rDNA was amplified by PCR. The primers 341F (CCT ACG GGNGGCWGC AG) and 806R (GGA CTA CHVGGG TAT CTAAT) were used to amplify the V3–V4 region of the 16S rRNA gene. The composition spectrum of intestinal microbial diversity was studied. The tags were clustered into different operational taxonomic units (OTUs) using Uparse software, and the sequence homology was 97% [24]. Shannon index was used to analyze the species diversity (α diversity), and UniFrac distance was used to calculate the β diversity of the samples. The linear discriminant analysis (LDA) effect size (LEfSe) was used to identify the significantly abundant taxa (phylum to genera) of bacteria among groups (LDA score > 2, *p* < 0.05).

### Statistical analysis

The data are expressed as the mean ± standard deviation. Analysis was performed using GraphPad Prism 9.3.0 software, and a two-tailed *p*-value < 0.05 was considered statistically significant. To determine significance among these groups, the Kruskal–Wallis test was used for non-normally distributed data analysis, and one-way ANOVA followed by T ukey’s test was used for normally distributed data. Six microphotographs per group were used for area quantification in the immunofluorescence analysis, and at least three colon samples per group were used for western blot analysis.

## Results

### Protective effect of oral TS on UC

**Fig. 1:**
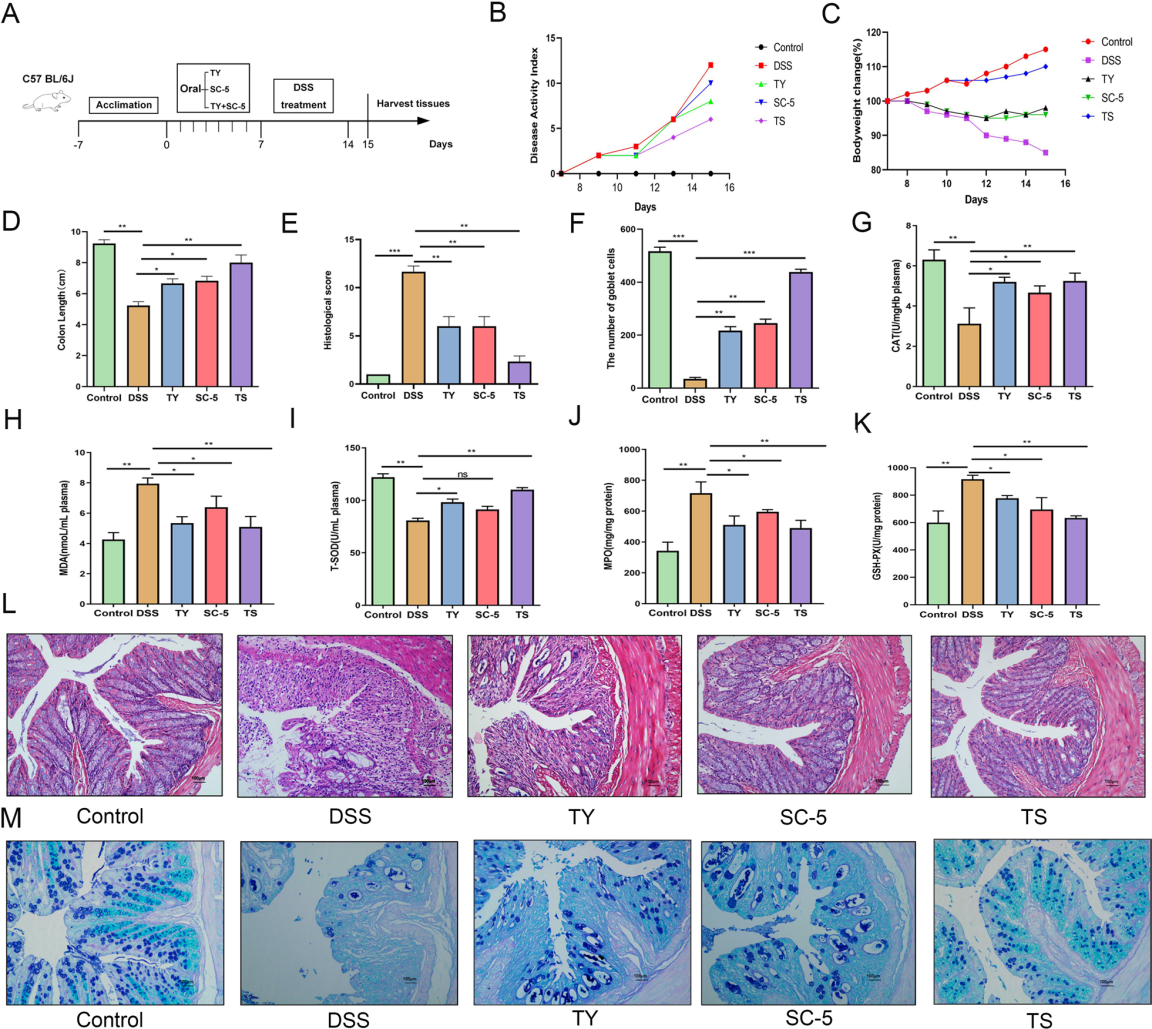
TS ameliorated the pathological symptoms of DSS-induced UC mice. **A** Experimental procedure; **B** disease activity index; **C** body weight changes; **D** colon length; **E** histopathology score; **F** the number of goblet cells; **G** CAT in plasma; **H** MDA in plasma; **I** T-SOD in plasma; **J** level of MPO in the colon; **K** level of GSH-PX in the colon; **L** representative H&E-stained colon sections images (magnification ×100); **M** representative AB-PAS strain colon sections images (magnification ×100). The data are expressed as the mean ± standard deviation (n = 9). Significance was determined by ANOVA with Tukey’s analysis, *p < 0.05, **p < 0.01, ***p < 0.001. (Control, DSS: dextran sodium sulfate; SC-5: *Lactobacillus plantarum* SC-5; TY: tyrosol; TS: *Lactobacillus plantarum SC-5* and tyrosol)

To investigate the palliative effect of TS on DSS-induced colitis, experimental colitis was induced in mice by continuous administration of 3% DSS for 7 days, followed by daily oral administration of SC-5, TY, or TS (Fig. 1A), based on our previously observed optimal dose (data not shown). As shown in Fig. 1B, DSS administration decreased body weight in all groups of mice compared with the normal group. However, compared with the DSS group, the SC-5, TY, and TS groups were protective against DSS treatment for weight loss, with TS being the most protective against weight loss (Fig. 1C). Among them, oral TS intervention significantly alleviated DSS-induced colitis, significantly reduced the disease activity index (DAI, a composite score of weight loss, stool consistency, and rectal bleeding), and colon shortening (Fig. 1D) (*p* < 0.05).

The results of H&E staining showed that DSS treatment resulted in severe colonic edema, loose epithelial structure, massive inflammatory cell infiltration and goblet cell loss, while oral administration of SC-5, TY, and TS improved the histopathological injury. In Fig. 1E, L, it can be found that compared with the DSS group, the structural integrity of epithelial cells in the colon tissue of the TY-treated group and the SC-5 treated group was improved to a certain extent, and the number of goblet cells was increased to a certain extent, but there was still some infiltration of inflammatory cells. The effect of TS was significantly better than that of TY and SC-5 alone. The structure of mucosa, submucosa, muscularis and serosa in TS group was compact and intact, and there were no exfoliated and necrotic epithelial cells in the intestinal lumen. The number of goblet cells in TS group was significantly higher than that in DSS group (*p* < 0.05), TY group and SC-5 group, and the infiltration of inflammatory cells was significantly reduced, even close to that in the control group. These indicate that the administration of TY and SC-5 alone can alleviate the histopathological damage of DSS-induced colitis in mice to a certain extent. TS had a more significant protective effect on colitis in mice compared with TY and SC-5 alone.

AB-PAS staining is shown in Fig. 1F. After DSS treatment, the mucosal layer of the colon was significantly thinned and a large number of goblet cells were lost, showing more severe pathological damage. However, TY, SC-5 and TS significantly restored the thickness and integrity of the colonic mucosa, and the number and integrity of goblet cells were higher, even close to the control group, showing a good protective effect on the colon. TS and SC-5 have a good effect on restoring the number of goblet cells and the thickness of intestinal mucosa.

### Oral administration of TS can decrease the level of pro-inflammatory cytokines and suppress oxidative stress injury

**Fig. 2:**
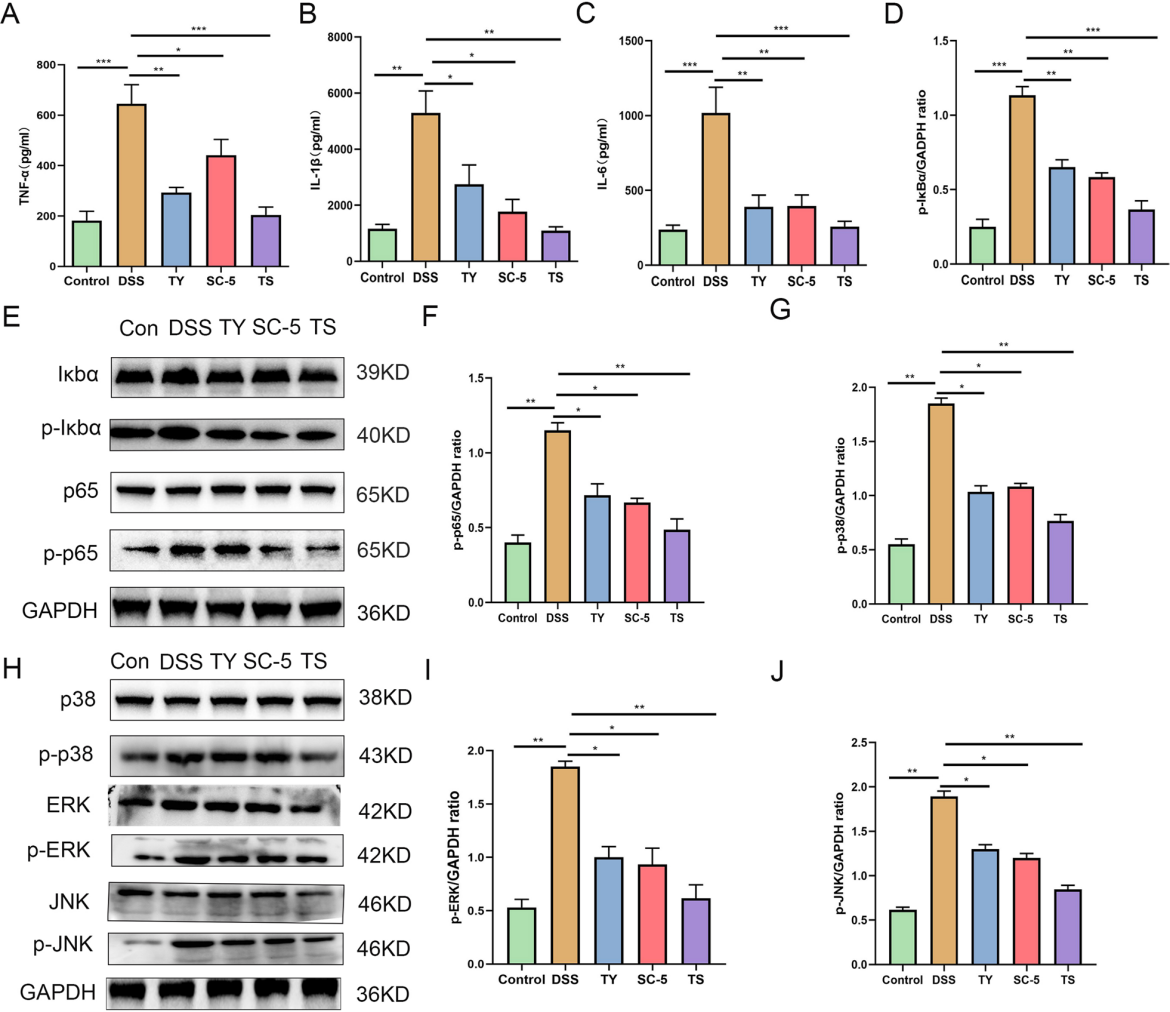
TS alleviated inflammatory pathway in the DSS-induced UC mice. **A** The inflammatory cytokines level of TNF-α; **B** IL-1β; **C** IL-6; **D** the relative expression of IκBα phosphorylation levels was normalized to GAPDH; **E** p65 and IκBα phosphorylation levels were analyzed by Western blot; **F** the relative expression of p65 phosphorylation levels was normalized to GAPDH; **G** the relative expression of p38 phosphorylation levels was normalized to GAPDH; **H** MAPK p38, JNK, and ERK phosphorylation levels were analyzed by Western blot; **I** the relative expression of ERK phosphorylation levels were normalized to GAPDH; **J** the relative expression of JNK phosphorylation levels were normalized to GAPDH. The data are expressed as the mean ± standard deviation (n = 6). Significance was determined by ANOVA with Tukey’s analysis, *p* < 0.05, **p < 0.01, ***p < 0.001. (Control, DSS: dextran sodium sulfate; SC-5: *Lactobacillus plantarum* SC-5; TY: tyrosol; TS: *Lactobacillus plantarum SC-5* and tyrosol)

In order to further explore the specific mechanism of TS alleviating colitis in mice. we examined inflammatory cytokines TNF-α, IL-1β, and IL-6 (Fig. 2A–C), and oxidative stress related index MPO, T-SOD, CAT, T-AOC, GSH-PX, and MDA in the colonic tissue and blood of mice (Fig. 1G–K). Compared with the control group, DSS treatment led to a significant increase in the concentrations of TNF-α, IL-1β and IL-6, the concentrations of MPO and MDA significantly increase and T-SOD, CAT, T-AOC, and GSH-px significantly decrease (*p* < 0.05). However, the levels of pro-inflammatory cytokines in the serum and colonic tissue of mice treated with SC-5, TY, or TS were significantly decreased, the contents of MPO and MDA were significantly reduced to levels similar to those in the control group, and the enzyme activities of T-SOD, CAT, T-AOC, and GSH-PX were significantly increased (*p* < 0.05). The above results indicate that the protective effect of TS on UC is superior to that of TY and SC-5 groups (*p* < 0.05). The results indicate that TS can improve the activity of antioxidant stress-related enzymes in vivo, alleviate the oxidative stress injury of the body, and then alleviate the DSS-induced colitis in mice.

### Oral administration of TS can inhibit the activation of NF-κB and MAPK signaling pathways

NF-κB and MAPK signaling pathways are the main inflammation-related signaling pathways in UC. To explore the mechanism of protective effect of TS on colonic inflammation, we examined the levels of several key proteins in NF-κB and MAPK signaling pathways. As shown in Fig. 2E, the phosphorylation levels of NF-κB p65 and IκBα were upregulated after DSS intake, while the levels of SC-5, TY, and TS groups significantly downregulated the phosphorylation levels of NF-κB p65 and IκBα compared with DSS group (*p* < 0.05), indicating that the activation of NF-κB signaling pathway was inhibited.

Over-activation of MAPK has been shown to be involved in the progression of UC, and MAPK is an important pathway regulating inflammation. As shown in Fig. 2H, the MAPK pathway was significantly activated in the DSS-treated group, while the phosphorylation levels of p38, ERK, and JNK were significantly decreased in the SC-5, TY, and TS groups (*p* < 0.05).

We found that the oral administration of TS had the best inhibitory effect on NF-κB and MAPK signaling pathway activation among three different administrations of SC-5, TY, and TS. These data suggest that the ameliorating effect of TS on colonic inflammation in mice is related to the inhibition of NF-κB and MAPK pathways, this is consistent with the findings of previous studies [26, 27].

### Oral administration of TS enhances the integrity of the intestinal mucosal barrier

**Fig. 3:**
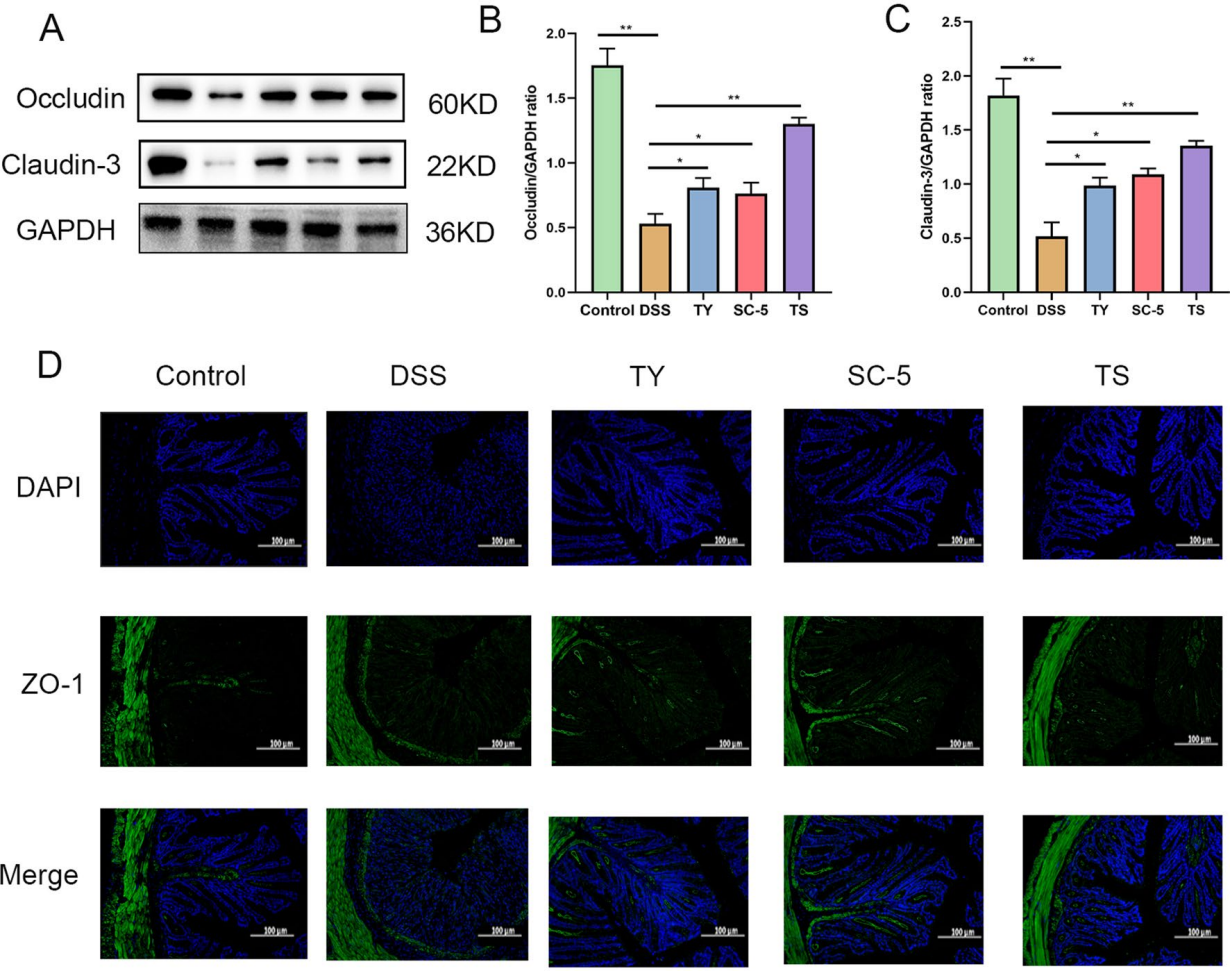
TS alleviated gut barrier damage in the DSS-induced UC mice. **A** Protein expression of Occludin and Claudin-3 in the five groups determined using Western blot. **B** The relative expression of Occludin was normalized to GAPDH; **C** the relative expression of Claudin-3 was normalized to GAPDH; **D** representative images of Control, DSS, S5, TY and TS at the level of ZO-1 by immunofluorescence staining. The image was taken by a confocal microscope with ×100 magnification. Blue: DAPI in the nucleus; Green: ZO-1 in the colon. The data are expressed as the mean ± standard deviation (n = 6). Significance was determined by ANOVA with Tukey’s analysis, *p* < 0.05, **p < 0.01, ***p < 0.001. (Control, DSS: dextran sodium sulfate; SC-5: *Lactobacillus plantarum* SC-5; TY: tyrosol; TS: *Lactobacillus plantarum SC-5* and tyrosol)

TJ protein is a kind of key protein connecting the intercellular space and regulating the permeability of intestinal mucosa. TJ mainly include Occludins, Zonula occludens (ZO) and claudin family proteins. They facilitate the formation of intercellular connections by interacting with proteins that link the cell’s cytoskeleton to other cell’s tight junction proteins, thereby contributing to the formation of the intestinal mucosal barrier [28]. In patients with IBD, studies have found that the expression and function of ZO-1, Occludin, and Claudin-3 may be affected, leading to compromised intestinal mucosal barrier and increased permeability. This situation can result in the infiltration of intestinal microbes and toxins, triggering an inflammatory response, thereby exacerbating the progression of the disease and worsening symptoms [29]. Pro-inflammatory cytokines such as IL-6, IL-1β and TNF-α can decrease the expression TJ protein, thereby enhancing intestinal permeability and aggravating intestinal barrier dysfunction [30]. To further explore the protective effect of TS on DSS-induced UC, we examined the expression of ZO-1, Occludin, and Claudin-3 (Fig. 3). As expected, DSS treatment significantly reduced the expression of ZO-1, Occludin, and Claudin-3 proteins (*p* < 0.05). The expression of ZO-1, Occludin and Claudin-3 was significantly increased in SC-5, TY and TS groups (*p* < 0.05). These results suggest that SC-5, TY and TS can enhance the expression of TJ proteins in the colon of mice with DSS-induced colitis, thereby restoring the integrity of the intestinal mucosal barrier. Increased expression of TJ proteins can enhance the tight connection between cells, thus enhancing the intestinal barrier effect and alleviating UC symptoms.

**Fig. 4:**
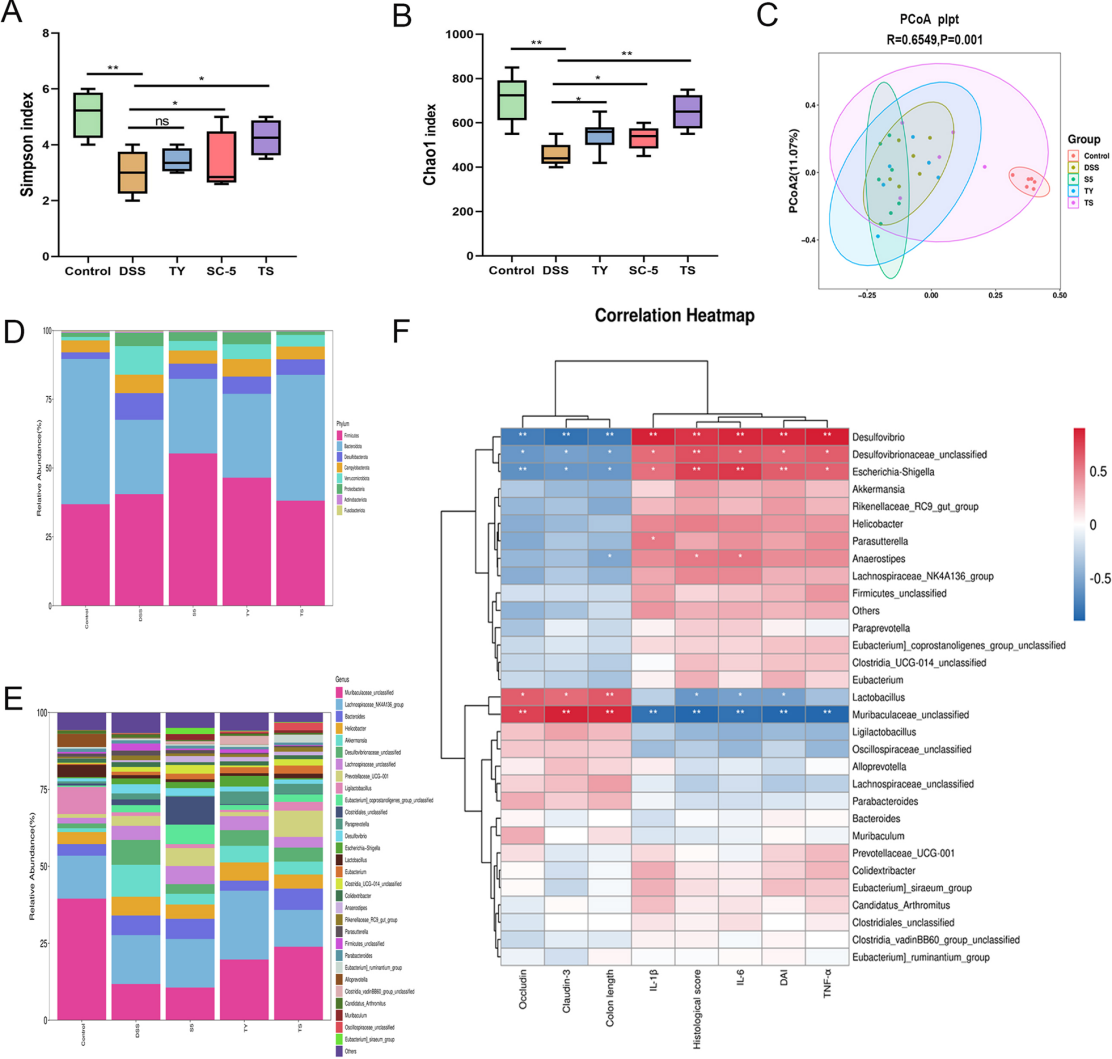
Effects of TS supplementation on the diversity and composition of gut microbiota. **A** The α-diversity of gut microbiota was determined using Simpson index; **B** the α-diversity of gut microbiota was determined using Chao1 index; **C** unweighted UniFrac-based principal coordinate analysis (PCoA) based on the OTU levels; **D** the flora composition of mice at the phylum level (Additional file 1: Table S1); **E** the flora composition of mice at the genus level (Additional file 2: Table S2); **F** correlation heatmap analysis using the Spearman correlation coefficient to determine the relationship between intestinal bacteria and the severity of Colon length, DAI score, Histopathology score, IL-β, IL-6, TNF-α, Occludin, and Cluadin-3. The data are expressed as the mean ± standard deviation. ns, not significant; *p* < 0.05, **p < 0.01, ***p < *0.001.* (Control, DSS: dextran sodium sulfate; SC-5: *Lactobacillus plantarum* SC-5; TY: tyrosol; TS: *Lactobacillus plantarum SC-5* and tyrosol)

Oral administration of TS can regulate the composition of intestinal microbiota. It was found that the microbial richness, evenness, and diversity of the DSS group were significantly reduced by examining the cecal contents of mice, while the species diversity, richness, and community evenness could be effectively restored by supplementing TY (*p* < 0.05). The structural composition of the intestinal microbiota at the phylum and genus level in the five groups (Control, DSS and TY, SC-5, TS) is shown in Fig. 4. By contrast, the relative abundance of DSS-group *Bacteroides* and *Proteus* increased, but the relative proportion of *Actinobacteria* and *Firmicutes* decreased. *Firmicutes/Bacteroides* ratio can be used as a biomarker for intestinal inflammation [31]. The ratio of *Firmicutes*/*Bacteroides* rose gradually with the supplementation of TY, SC-5, and TS. *Bifidobacteria*, *Rikenellacee_RC9_gut_group*, *Akkermannia*, *Rhamnaceae*, *Lactobacillus*, and *Alitipes* in the TS group returned to the levels of the control group compared with the DSS, SC-5, TS group (Fig. 4).

Spearman correlation analysis was further performed to understand the relationship between differentially enriched microorganisms and antioxidant, inflammation parameters or SCFAs profiles. Correlation analysis showed a significant positive correlation between *Akkermansia* and CAT levels in plasma (*p* < 0.05) and a significant negative correlation between *Akkermansia* and IL-6 levels in colon (*p* < 0.05) (Fig. 4). However, lactic acid bacteria enriched in the colitis group showed a strong inverse correlation with colonic TNF-α, IL-1β,and IL-6 levels (*p* < 0.05). Most importantly, oral administration of TS caused alterations in the intestinal microbiota in mice with colitis, suggesting that the intestinal microbiota may play a key role in alleviating DSS-induced colitis.

### TS–FMT relieves clinical symptoms of colitis better than CON–FMT

**Fig. 5:**
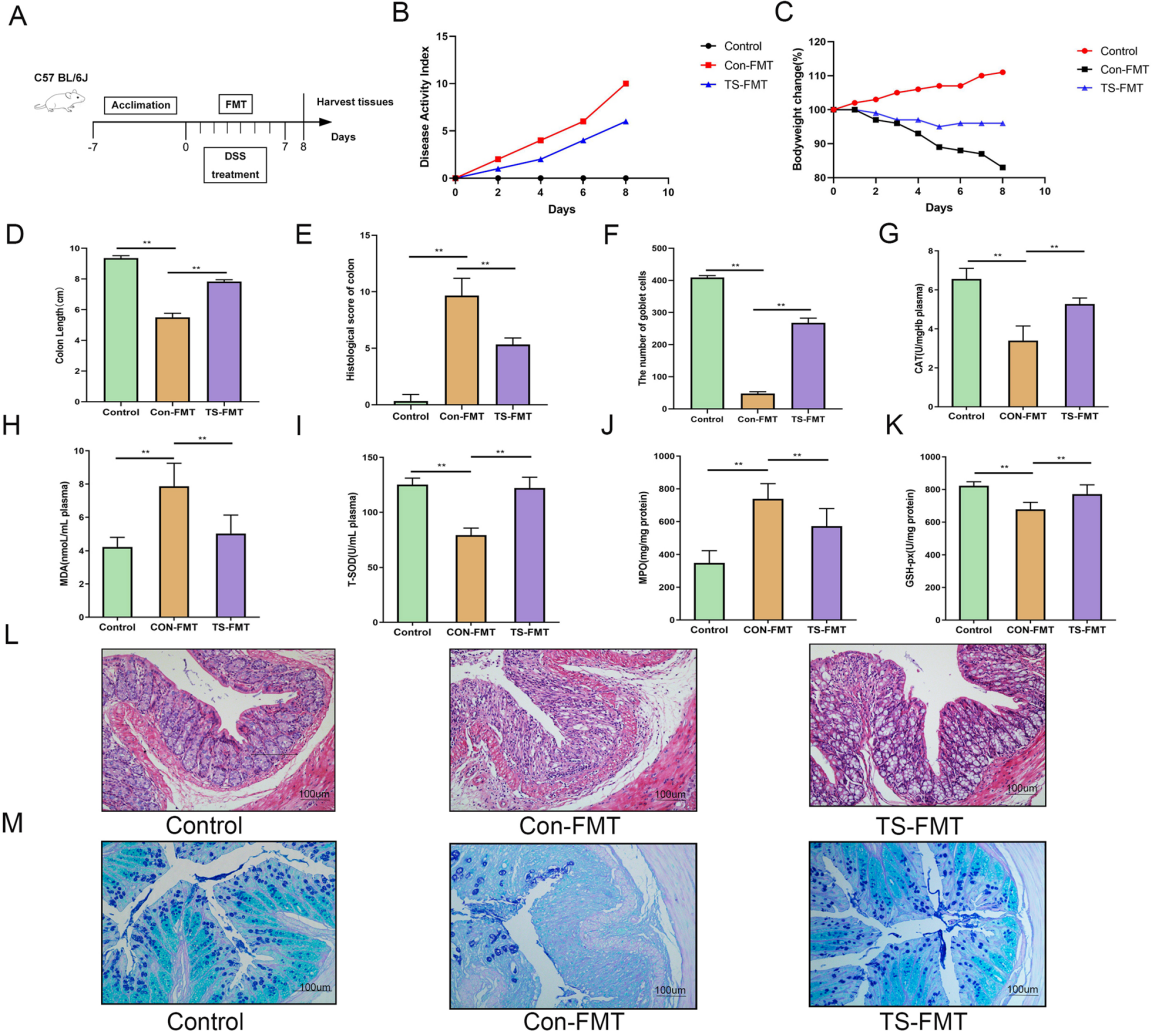
TS–FMT ameliorated the pathological symptoms of DSS-induced UC mice. TS ameliorated the pathological symptoms of DSS-induced UC mice. **A** Experimental procedure; **B** disease activity index; **C** body weight changes; **D** colon length; **E** histopathology score; **F** the number of goblet cells; **G** CAT in plasma; **H** MDA in plasma; **I** T-SOD in plasma; **J** level of MPO in the colon; **K** level of GSH-PX in the colon; **L** representative H&E-stained colon sections images (magnification ×100); **M** representative AB-PAS strain colon sections images (magnification ×100). The data are expressed as the mean ± standard deviation (n = 9). Significance was determined by ANOVA with Tukey’s analysis, *p* < 0.05, **p < 0.01, ***p < 0.001. (CON–FMT: the feces of the Control group were given to the DSS-treated mice for fecal microbiota transplantation, TS–FMT: the feces of the normal mice orally administered with TS alone were given to the DSS-treated mice for fecal microbiota transplantation)

We next validated the effect of TS-mediated microbiota on murine colitis in a second animal assay by transplanting fecal microbiota from mice receiving TS gavage to DSS-treated mice (Fig. 5A). Compared with CON–FMT, TS–FMT treated mice with colitis showed less reduction in body weight and colon length, and less DAI score and histopathological damage (Fig. 5B). Levels of pro-inflammatory factors such as IL-1β, IL-6, and TNF-α were significantly decreased in serum, IL-6 and TNF-α levels were significantly decreased in colon, and the activity of MPO, an oxidative stress-related enzyme, was significantly increased in TS–FMT-treated colitis mice compared with the other three groups (Fig. 5B) (*p* < 0.05). The results showed that the treatment of TS can significantly inhibit UC induced by DSS, but in clinical treatment, the therapeutic effect of drugs made from fecal extracts and direct fecal transplantation on UC is unknown.

### Activation of NF-κB and MAPK signaling access was more inhibited by TS–FMT than by CON–FMT

**Fig. 6:**
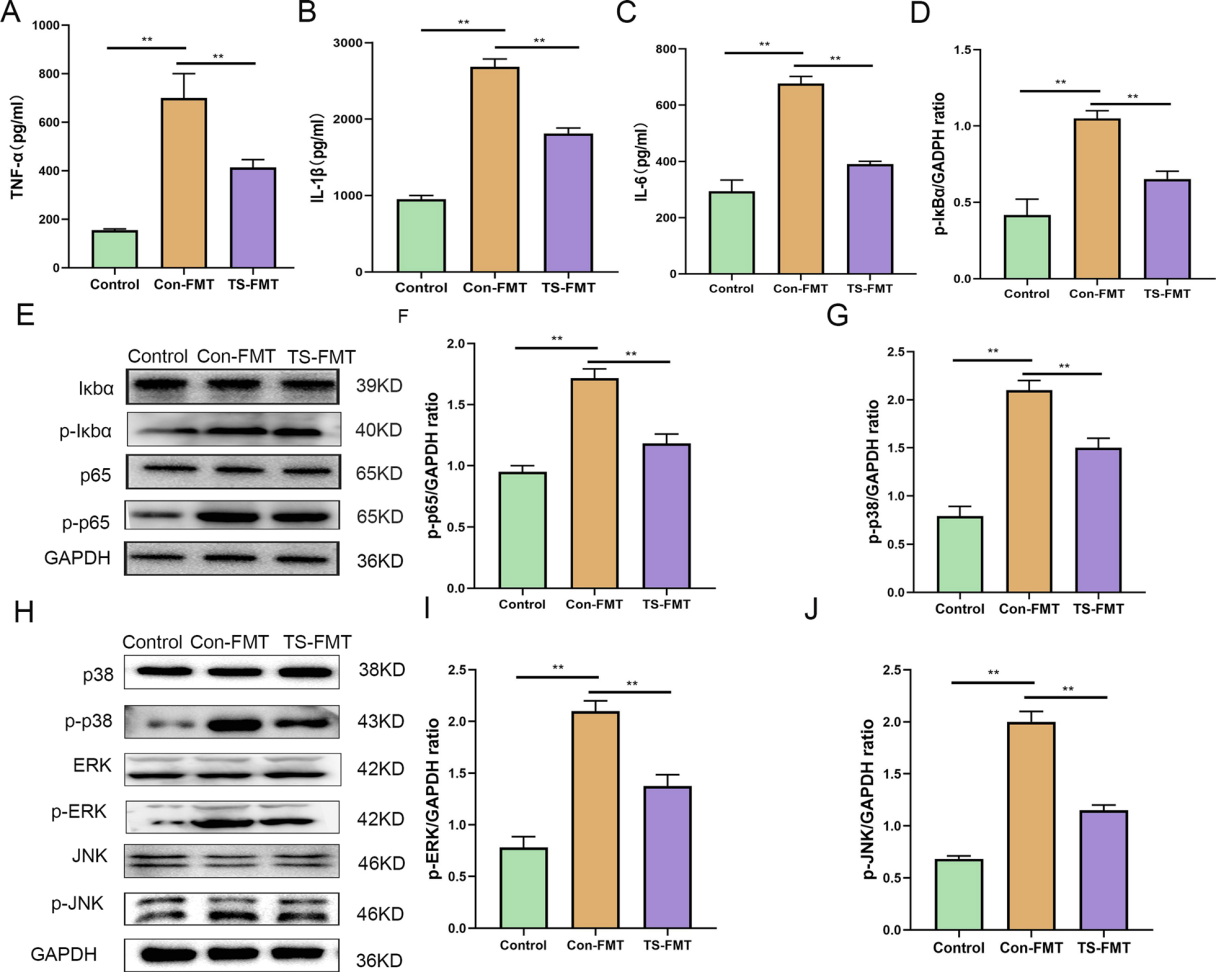
TS–FMT alleviated inflammatory pathway in the DSS-induced UC mice. **A** The inflammatory cytokines level of TNF-α; **B** IL-1β; **C** IL-6; **D** the relative expression of IκBα phosphorylation levels was normalized to GAPDH; **E** p65 and IκBα phosphorylation levels were analyzed by Western blot; **F** the relative expression of p65 phosphorylation levels was normalized to GAPDH; **G** the relative expression of p38 phosphorylation levels was normalized to GAPDH; **H** MAPK p38, JNK, and ERK phosphorylation levels were analyzed by Western blot; **I** the relative expression of ERK phosphorylation levels were normalized to GAPDH; **J** the relative expression of JNK phosphorylation levels were normalized to GAPDH. The data are expressed as the mean ± standard deviation (n = 6). Significance was determined by ANOVA with Tukey’s analysis, *p* < 0.05, **p < 0.01, ***p < 0.001. (CON–FMT: the feces of the Control group were given to the DSS-treated mice for fecal microbiota transplantation, TS–FMT: the feces of the normal mice orally administered with TS alone were given to the DSS-treated mice for fecal microbiota transplantation)

Compared with the normal group, CON–FMT observed that the phosphorylation levels of p-p65 and p-IκBα were significantly increased, and the expressions of p-p38, p-JNK and p-ERK were also significantly up-regulated in colitis mice (*p* < 0.05), indicating that NF-κB and MAPK access were over-activated (Fig. 6E–J). The inhibitory effect of CON–FMT on DSS-induced activation of the inflammatory signal access was not evident. Compared with CON–FMT group, NF-κB and MAPK access activation were significantly inhibited, and the expressions of p-p65, p-IκBα, p-p38, p-JNK and p-ERK were significantly down-regulated in TS–FMT group (*p* < 0.05). These results suggest that TS–FMT is more effective than CON–FMT in inhibiting DSS-induced activation of colitis-associated Inflammation signaling access in mice.

### Compared with CON–FMT, TS–FMT enhanced the expression of tight junction protein in intestinal mucosa

**Fig. 7:**
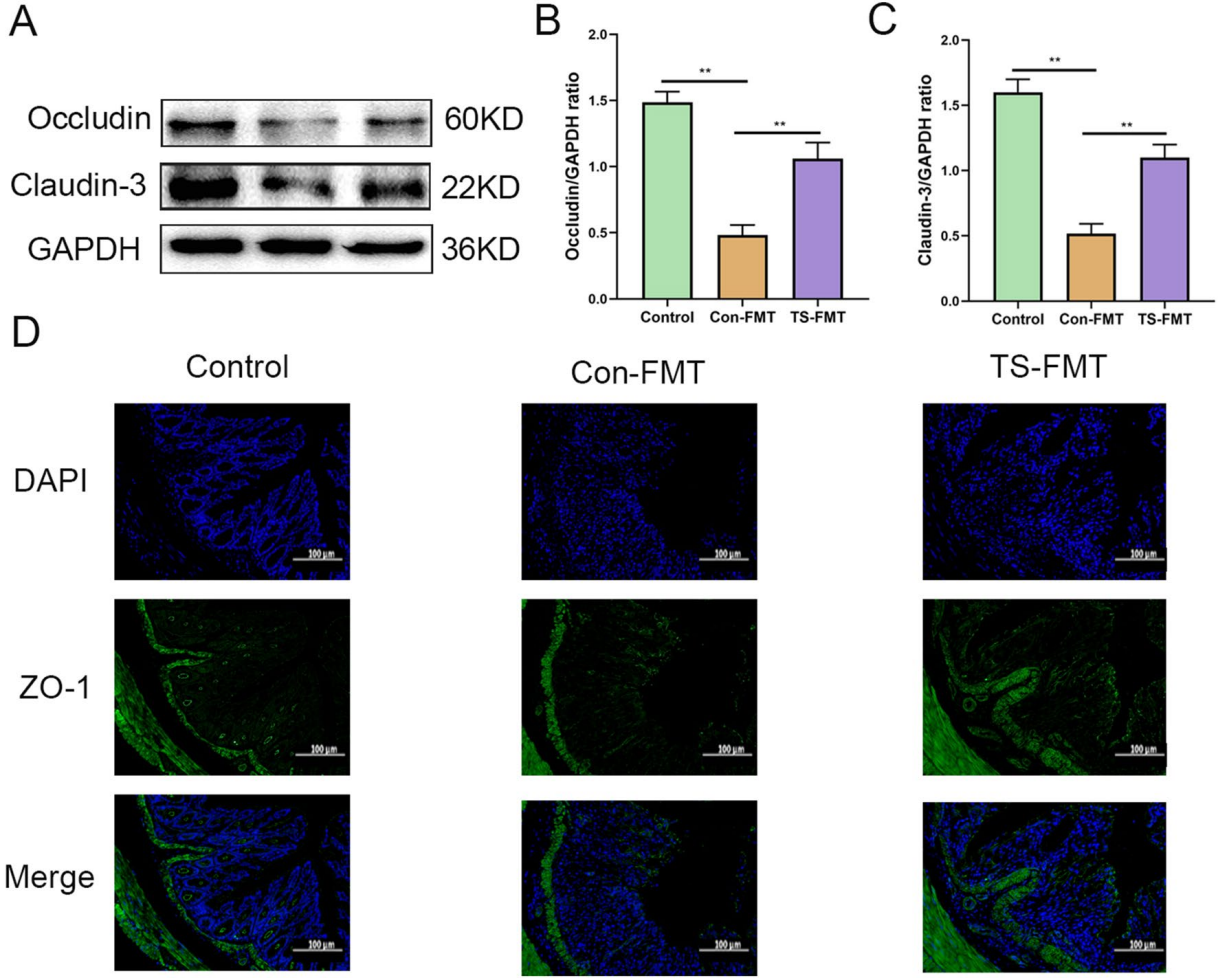
TS–FMT alleviated gut barrier damage in the DSS-induced UC mice. **A** Protein expression of Occludin and Claudin-3 in the three groups determined using Western blot; **B** the relative expression of Occludin was normalized to GAPDH; **C** the relative expression of Claudin-3 was normalized to GAPDH; **D** representative images of Control, DSS, S5, TY and TS at the level of ZO-1 by immunofluorescence staining. The image was taken by a confocal microscope with ×100 magnification. Blue: DAPI in the nucleus; Green: ZO-1 in the colon. The data are expressed as the mean ± standard deviation (n = 3). Significance was determined by ANOVA with Tukey’s analysis, *p* < 0.05, **p < 0.01, ***p < 0.001. (CON–FMT: the feces of the Control group were given to the DSS-treated mice for fecal microbiota transplantation, TS–FMT: the feces of the normal mice orally administered with TS alone were given to the DSS-treated mice for fecal microbiota transplantation)

According to the WB and immunofluorescence results (Fig. 7), we found that the expression of tight junction proteins Occludin, Claudin-3, and ZO-1 in CON–FMT was significantly down-regulated, and the loss of tight junction proteins was severe, which was significantly reversed by TS–FMT. The expression of Occludin, Claudin-3, and ZO-1 was higher than that of CON–FMT (*p* < 0.05), and even recovered to the level close to the normal control group. These results indicate that TS–FMT can effectively up-regulate the expression of tight junction proteins in intestinal mucosa, repair the damaged intestinal mucosal barrier and restore its integrity.

### TS–FMT regulates intestinal microbiota structure better than CON–FMT

**Fig. 8:**
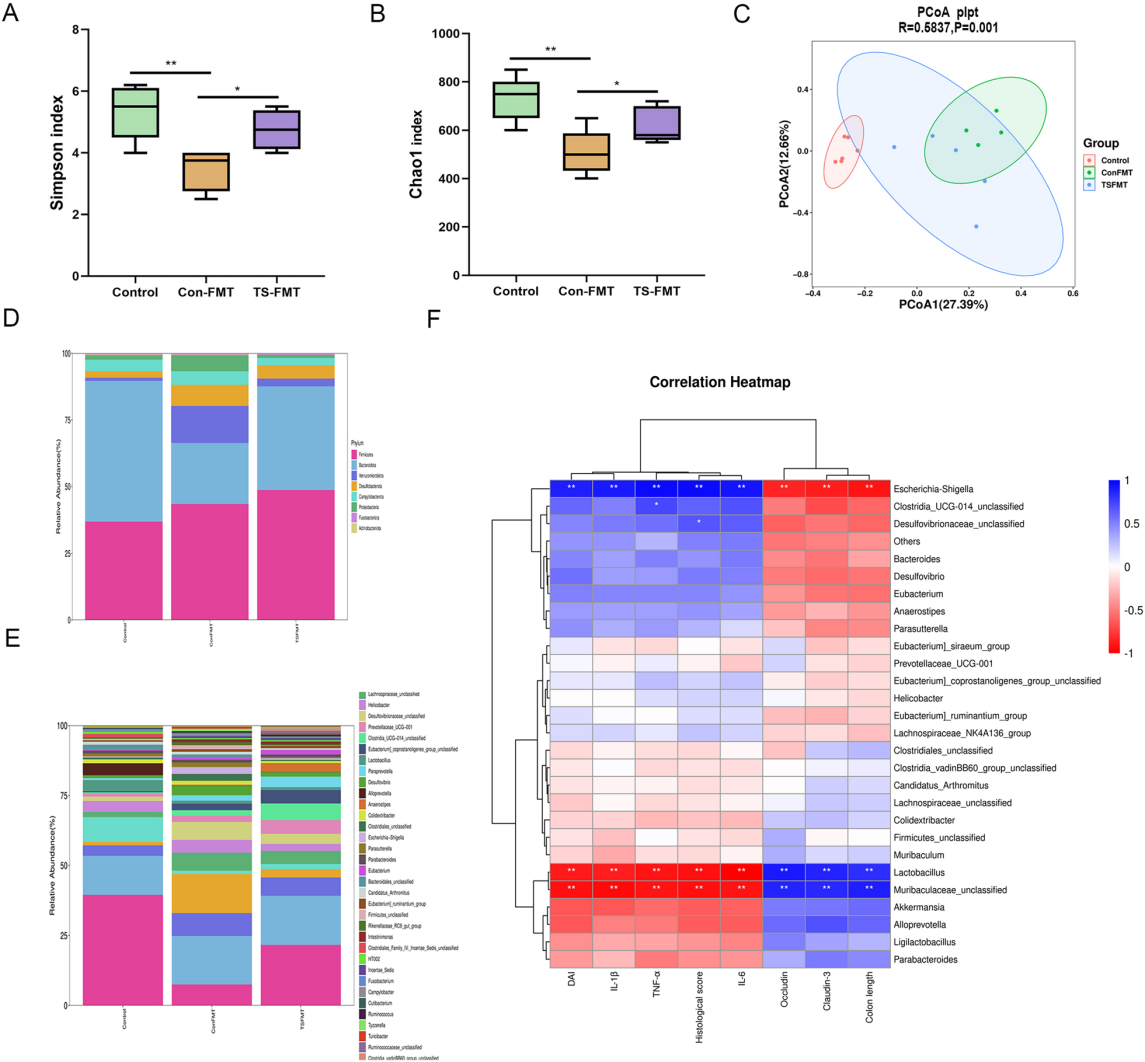
Effects of TS–FMT supplementation on the diversity and composition of gut microbiota. **A** The α-diversity of gut microbiota was determined using Simpson index; **B** the α-diversity of gut microbiota was determined using Chao1 index; **C** unweighted UniFrac-based principal coordinate analysis (PCoA) based on the OTU levels; **D** the flora composition of mice at the phylum level (Additional file 3: Table S3); **E** the flora composition of mice at the genus level (Additional file 4: Table S4); **F** correlation heatmap analysis using the Spearman correlation coefficient to determine the relationship between intestinal bacteria and the severity of Colon length, DAI score, Histopathology score, IL-β, IL-6, TNF-α, Occludin, and Cluadin-3. The data are expressed as the mean ± standard deviation (n = 6). *p* < 0.05, **p < 0.01, ***p < *0.001.* (CON–FMT: the feces of the Control group were given to the DSS-treated mice for fecal microbiota transplantation, TS–FMT: the feces of the normal mice orally administered with TS alone were given to the DSS-treated mice for fecal microbiota transplantation)

According to the results of 16s rRNA, CON–FMT significantly increased the abundance of *Helicobacter*, *Desulfovibrionaceae, Clostridiale* and *Escherichia* and decreased the abundance of *Ligilactobacillus* and *Muribaculaceae* (Fig. 8) (*p* < 0.05). However, these results were significantly reversed by TS–FMT. TS–FMT significantly restored the abundance of *Ligilactobacillus* and *Muribaculaceae*, and reduced the abundance of *Helicobacter*, *Desulfovibrionaceae*, *Clostridiale* and *Escherichia*, and significantly increased alpha and beta diversity (*p* < 0.05). *Desulfovibrio* and Escherichia were positively correlated with IL-1β, IL-6, TNF-α, histopathologic score and DAI score. And negatively correlated with Claudin-3, Occludin and colon length. *Muribaculaceae* and *Lactobacillus* were negatively correlated with IL-1β, IL-6, TNF-α and histopathologic score and DAI score. While positively correlated with Claudin-3, Occludin and colon length.

## Discussion

UC, a type of IBD, has become a global health challenge and is currently on the rise [32, 33]. Studies have shown that oxidative stress may be an important factor in exacerbating UC, which can lead to neutrophils to produce ROS and other substances. Excessive production of ROS will lead to oxidative stress injury of the body and worsen the symptoms of UC [34, 35]. Previous studies have suggested that probiotics or prebiotics may be effective by regulating intestinal microbiota, and probiotics and may reduce ROS production by regulating the structure of microbial intestinal microbiota [36]. In previous studies, we found that *Lactobacillus plantarum* SC-5 and an olive oil extract TY can reduce the level of oxidative stress in ulcerative colitis mice. According to research reports, combining natural antioxidant products with probiotics has shown a stronger effect in alleviating UC symptoms [17, 18, 37]. Therefore, in order to reduce this oxidative stress, we chose *Lactobacillus plantarum* SC-5 as a new probiotic strain and used TY, as a unique natural prebiotic source to explore its therapeutic effect on UC.

In order to evaluate the protective effect of TS on DSS-induced UC, it was applied to in vivo experiments. The DSS-induced colitis model is an important tool to assess the pathogenesis of UC, which is similar to the human UC pathogenesis [38]. DAI is widely used to evaluate the health of UC models [39]. The DAI score, which is composed of weight change, fecal viscosity and hematochezia in mice, plays an important role in evaluating UC models [40]. Although the DSS-induced colitis mouse model is widely used in experiments, it has limitations in simulating systemic diseases because it mainly focuses on colonic inflammatory responses and cannot comprehensively simulate the systemic features of human UC. Additionally, the model cannot fully replicate the complex pathophysiological processes of human UC. Despite the limitations of animal models, drug research remains a crucial step in understanding diseases in depth and improving patients’ quality of life. Therefore, research on colitis drugs remains of significant importance. While the mouse model is an important tool for studying UC, further research and clinical validation are still required to translate drugs from mouse models to human treatments.

Oral administration of SC-5, TY, and TS resulted in a reduced degree of body weight loss and a reduced length of colon shortening compared to the DSS group, indicating that SC-5, TY and TS were able to restore the apparent clinical symptoms of DSS-induced colitis in mice. In this study, the concentrations of MPO and MDA in DSS group were significantly increased, while the activities of T-SOD, T-AOC, GSH-PX and CAT were significantly decreased, while SC-5, TY, and TS groups significantly reversed these changes, indicating that TS can alleviate oxidative stress damage in the process of colonic inflammation. It has a protective effect on the colon of mice.

DSS-mediated UC produces large amounts of pro-inflammatory cytokines, such as TNF-α, IL-1β, and IL-6 in the serum and colonic tissue of patients [41, 42]. TNF-α, IL-1β and IL-6 play a crucial role in the pathogenesis of IBD [43]. In the early stage of inflammation, a large amount of TNF-α can be produced, and the increase of TNF-α can destroy the tight structure of intestinal epithelium [44]. The cytokines IL-1β secreted by macrophages is involved in the pro-inflammatory response that promotes the progression of UC [45]. IL-6 is produced by lymphocytes produced by activated T cells and fibroblasts and mediates the process of inflammation [46]. In this study, after DSS treatment, the pro-inflammatory cytokines was significantly increased, indicating that the inflammation response was enhanced, and under the protection of SC-5,TY, and TS, the pro-inflammatory cytokines level was significantly decreased, indicating that the inflammation level in the body was inhibited. This suggests that TS has an ameliorative effect on UC, which may be achieved by inhibiting the level of pro-inflammatory factor.

To further explore the mechanism by which TS-inhibited UC, we examined the expression of NF-κB and MAPK signaling access associated proteins, which play an important role in promoting inflammation cytokines production [47, 48]. NF-κB and MAPK are cellular access that mainly promote the production of inflammation and have a tremendous impact on the production of cytokines. In our previous study, we measured the changes of pro-inflammatory factors in serum and colonic tissue of colitis mice, and found that SC-5, TY, and TS could inhibit the activation of related inflammation signaling access. To verify whether this change was mediated by NK-κB and MAPK access, we examined the expression of NF-κB and MAPK related proteins. As shown in the results, SC-5, TY, and TS all inhibited the activation of NF-κB and MAPK signaling access. DSS treatment significantly increased the expression of p-p65, p-IκBα, p-p38, p-JNK and p-ERK, which are involved in the activation of NF-κB signaling access, and DSS treatment also increased the expression of p-p38, p-JNK and p-ERK, which are involved in the activation of MAPK access signaling. These results suggest that the anti-UC effects of SC-5, TY and TS are mediated by the inhibition of NF-κB and MAPK activation. Taken together, the NF-κB and MAPK pathways play important roles in the pathogenesis of UC. Their abnormal activation may lead to sustained and intensified inflammatory responses, thereby exacerbating symptoms and disease progression in UC patients. Therefore, targeting the regulation of these two pathways may become an important strategy for treating UC and developing new therapeutic products for UC.

The intestinal barrier refers to the physiological barrier on the surface of the intestinal mucosa, which is composed of mucosal cell layers, mucus layer, and intestinal epithelial cells. It plays an important role in preventing the invasion of harmful substances, regulating immune responses, and maintaining the balance of the intestinal microbiota. Epithelial TJ proteins are essential for the formation of the cytoskeleton and maintenance of the intestinal mechanical barrier [49]. It has been reported that the expression of Occludin, Claudin-3 and ZO-1 proteins in TJ proteins was significantly decreased in UC patients [50]. Therefore, we analyzed Occludin, Claudin-3, and ZO-1 proteins by western blot and immunohistochemistry, and found that SC-5, TY and TS treatment restored the DSS-induced reduction of intestinal mucosal TJ protein expression. These results suggested that SC-5, TY, and TS may enhance the expression of TJ protein and then repair that mechanical barrier of the intestinal epithelium, thereby reducing the damage of the colon. Medications aimed at restoring intestinal barrier function may become part of the treatment strategy, thereby reducing inflammation and maintaining a stable microbial environment within the intestines.

The structure of intestinal microbiota plays an important role in the pathogenesis of UC, and different microorganisms are involved in pro-inflammatory or anti-inflammatory processes [51]. The *Bifidobacterium* can protect the integrity of the intestinal mucosa, reduce intestinal mucosal damage, and thus play a positive role in combating the development of UC [52]*. Muribaculaceae* can inhibit the activation of CD8+ T cells to tolerate immune stimulation and is negatively correlated with inflammation status [53]. *Lactobacillus* can regulate the balance of intestinal microbiota, reduce the growth of harmful bacteria, increase the number of beneficial bacteria, and have a positive impact on maintaining intestinal health [54]. In this study, we found that SC-5, TY, and TS increased the number of *Bifidobacterium*, and *Lactobacillus*. On the other hand, SC-5, TY, and TS reduced the number of *Proteobacteria*. It is reported that *Proteobacteria* is considered to be the harmful bacteria in the process of UC, which promotes the production of pro-inflammatory factors in the intestine [45, 46]. The observations of the present study indicate an increase in the relative abundance of bacteria and *Proteobacteria* in the DSS group compared to the control group, which is consistent with previous reports [55]. *Akkermansia* abundance has been shown to be positively associated with colorectal cancer [56, 57]. The UC model used in this study was consistent with the changes described above, with a significant reduction in *Akkermansia* in the cecal contents of mice in the DSS group, while oral administration of SC-5, TY and TS significantly reduced the abundance of *Akkermansia*, indicating that SC-5, TY, and TS have a positive effect on inhibiting the reproduction of *Akkermansia* in the intestine, which is of great significance for reducing the risk of colorectal cancer. DSS exposure altered the gut microbiota, and we observed an increase in the relative abundance of *Helicobacter*, *Bacteroides*, and *Shigella* in the DSS group, but they were suppressed by SC-5, TY, and TS. Increased abundance of *Bacteroides* may also be associated with the development and progression of UC, consistent with the findings of *Hartley*, who found high levels of *Bacteroides* in UC patients [58]. *Helicobacter* and *Shigella* belong to the phylum Proteus. *Helicobacter* is a potentially pathogenic bacterium that is positively correlated with inflammation [59]. The *Shigella* the most typical pathogen, may be involved in disrupting the intestinal mucosa and tight barrier junctions, leading to bleeding, which is consistent with other results of this study [60]. Taken together, our results indicate that DSS-induced changes in the gut microbiota can be restored by supplementation of SC-5, TY, and TS. The above results suggest that SC-5, TY, and TS reduced DSS-induced colitis by reducing the harmful microbiota and increasing the beneficial microbiota in the intestine. Although it has been demonstrated that SC-5, TY, and TS can regulate the intestinal microbiota and alleviate DSS-induced colitis, the specific mechanism by which SC-5, TY and TS alleviated UC and the specific functional components of its alteration remain to be further studied.

The interaction between the gut microbiota and the host’s innate immune system and adaptive immune system plays an important role in regulating intestinal homeostasis and inflammation. Some studies suggest that dysbiosis of the gut microbiota may exacerbate the pathological symptoms of UC [61]. In the three groups (SC-5, TY, and TS), TS showed better effects than the other two groups, so we will continue the dosage and mode of administration of TS in further experiments. Next, we sought to demonstrate whether TS is able to mediate the preponderant proliferation of probiotics in the gut and to verify whether the affected gut microbiota is a consequence or a cause of colitis remission. To establish the experimental colitis mouse model, the feces of the normal control group were given to the DSS-treated mice for FMT (CON–FMT) or the feces of the normal mice orally administered with TS alone were given to the DSS-treated mice for FMT (TS–FMT).

Interestingly, TS–FMT successfully alleviated acute colitis, and the therapeutic effect of TS–FMT was significantly better than that of CON–FMT, indicating that TS-mediated changes in intestinal microbiota play a leading role in alleviating colitis. TS–FMT significantly attenuated DSS-induced inflammation response and oxidative stress, which was similar to that of oral TS. Based on these results, we demonstrate that TS-mediated intestinal microbiota is responsible for the remission of UC. Combined with the significant effect of oral administration of TS on the microbial community in healthy mice and the successful palliative effect of TS–FMT, we propose that TS increases the low abundance of other microorganisms in normal controls, thereby ameliorating colitis.

We analyzed the structure of intestinal microbiota in mice by 16sr RNA and found that at the genus level, the relative abundance of *Muribaculaceae*, *Lachnospiraceae*, and *Lactobacillus* in the DSS treatment group was lower than that in control group, while these were increased in the TS–FMT group. *Muribaculaceae* belongs to *Bacteroidetes*, which can regulate immune cells to reduce the levels of pro-inflammatory cytokines [62]. In addition, compared with CON–FMT, TS–FMT enriches the abundance of *Clostridiales*, *Eubacterium* and other probiotics in the mouse intestine. Previous studies have shown that *Clostridiales* and *Eubacterium* are beneficial bacteria existing in the intestine [63]. These increased probiotics may play a vital role in the anti-inflammatory and antioxidant processes of TS. In our study, we also found that TS–FMT can reduce pro-inflammatory factors colon tissue of mice, inhibit the activation of NF-κB and MAPK signaling access, and strengthen tight junction proteins in intestinal mucosa to improve the integrity of colonic epithelium and maintain the homeostasis of colon [61, 62]. Through the fecal bacteria transplantation experiment, we can better understand the potential efficacy of probiotics in improving the symptoms of UC patients. This can help to develop more effective treatment strategies and bring better treatment results to patients. Experiments have confirmed that probiotic fecal bacteria transplantation plays an important role in regulating the balance of intestinal microbiota, which is particularly critical for patients with UC. Restoring the balance of intestinal microbiota may help reduce the inflammatory response and improve symptoms.

FMT experiment proved that TS could inhibit the occurrence of colonic inflammation in mice by regulating intestinal microbiota, which indicated that TS had the probiotic function of maintaining the balance of intestinal microbiota, increasing the abundance of beneficial bacteria and reducing intestinal inflammation. This further indicates that the imbalance of intestinal microbiota is closely related to the occurrence of UC. We conclude that TS can alleviate DSS-induced colitis in mice by regulating the structure of intestinal microbiota, increasing the abundance of beneficial bacteria and reducing the abundance of harmful bacteria. These results suggest that TS–FMT has a similar palliative effect to TS itself. Of note, TS–FMT significantly promoted the production of beneficial species, whereas CON–FMT did not, suggesting that the protective effect of TS in UC is dependent on probiotic species. Based on our results and previous studies, probiotic microorganisms may also play a beneficial role in the interaction between the gut microbiota and beneficial organisms to treat or prevent disease [57].

In view of the intestinal microbiota modulatory effect of TS administration on mice, it is believed that the beneficial effect of TS on the alleviation of UC progression mainly improves the DSS-induced intestinal microbiota structure in mice, implying a central role of the intestinal microbiota in mediating beneficial effects. These changes in the gut microbiota trigger significant antioxidant, anti-inflammatory, and barrier-enhancing processes that attenuate intestinal inflammation and damage. These findings provide new insights into TS-mediated remission of UC and will contribute to the development of therapeutic and preventive strategies for UC and other inflammatory diseases. In conclusion, TS can be considered as a potential prebiotic product to prevent or ameliorate UC. However, the clinical effect of TS on UC remains to be further studied.

## Conclusion

In conclusion, TY, SC-5, TS could regulate DSS-induced colitis in mice by associated with normal intestinal microbiota balance, thereby reducing the expression levels of pro-inflammatory factors IL-6 and IL-1β, TNF-α, correlated with the activation of NF-κB and MAPK signaling pathway, and maintaining the expression of intestinal mucosal epithelial tight junction proteins. However, based on animal studies, the exact mechanism and safety of TS need to be fully elucidated in further cellular research. These findings provide new insights into the treatment of UC in the disease process, and will help to develop treatment and prevention strategies for IBD and other inflammatory diseases. Before translating to human application, more preclinical research is needed, including in-depth evaluation of the drug’s efficacy, safety, and pharmacokinetics.

### Supplementary Information

**Additional file 1: Table S1.** The flora composition of mice at the phylum level of TS.

**Additional file 2: Table S2.** The flora composition of mice at the genus level of TS.

**Additional file 3: Table S3.** The flora composition of mice at the phylum level of TS-FMT.

**Additional file 4: Table S4.** The flora composition of mice at the genus level of TS-FMT.

## Data Availability

The datasets used and/or analyzed during the current study are available from the corresponding author on reasonable request.
